# New Cytotoxic 24-Homoscalarane Sesterterpenoids from the Sponge *Ircinia felix*

**DOI:** 10.3390/ijms160921950

**Published:** 2015-09-11

**Authors:** Ya-Yuan Lai, Li-Chai Chen, Chug-Fung Wu, Mei-Chin Lu, Zhi-Hong Wen, Tung-Ying Wu, Lee-Shing Fang, Li-Hsueh Wang, Yang-Chang Wu, Ping-Jyun Sung

**Affiliations:** 1 Graduate Institute of Marine Biology, National Dong Hwa University, Pingtung 944, Taiwan; E-Mails: spire0123456@yahoo.com.tw (Y.-Y.L.); jinx6609@nmmba.gov.tw (M.-C.L.); wanglh@nmmba.gov.tw (L.-H.W.); 2 National Museum of Marine Biology & Aquarium, Pingtung 944, Taiwan; 3 Department of Pharmacy of Zuoying Branch of Kaohsiung Armed Forces General Hospital, Kaohsiung 813, Taiwan; E-Mail: pharmacy@mail.ngh.com.tw; 4 Department of Marine Biotechnology and Resources, Asia-Pacific Ocean Research Center, National Sun Yat-sen University, Kaohsiung 804, Taiwan; E-Mail: wzh@mail.nsysu.edu.tw; 5 Division of Surgical Oncology, Department of Surgery, Kaohsiung Medical University Hospital, Kaohsiung 807, Taiwan; E-Mail: Hepatoma2003@yahoo.com.tw; 6 Chinese Medicine Research and Development Center, China Medical University Hospital, Taichung 404, Taiwan; E-Mail: kuma0401@gmail.com; 7 Department of Sport, Health and Leisure, Cheng Shiu University, Kaohsiung 833, Taiwan; E-Mail: lsfang@csu.edu.tw; 8 School of Pharmacy, College of Pharmacy, China Medical University, Taichung 404, Taiwan; 9 Center for Molecular Medicine, China Medical University Hospital, Taichung 404, Taiwan; 10 Graduate Institute of Natural Products, Kaohsiung Medical University, Kaohsiung 807, Taiwan

**Keywords:** *Ircinia felix*, sponge, homoscalarane, sesterterpenoid, cytotoxicity

## Abstract

Two new 24-homoscalarane sesterterpenoids, felixins F (**1**) and G (**2**), were isolated from the sponge *Ircinia felix*. The structures of new homoscalaranes **1** and **2** were elucidated by extensive spectroscopic methods, particularly with one-dimensional (1D) and two-dimensional (2D) NMR, and, by comparison, the spectral data with those of known analogues. The cytotoxicity of **1** and **2** against the proliferation of a limited panel of tumor cell lines was evaluated and **1** was found to show cytotoxicity toward the leukemia K562, MOLT-4, and SUP-T1 cells (IC_50_ ≤ 5.0 μM).

## 1. Introduction

**Figure 1:**
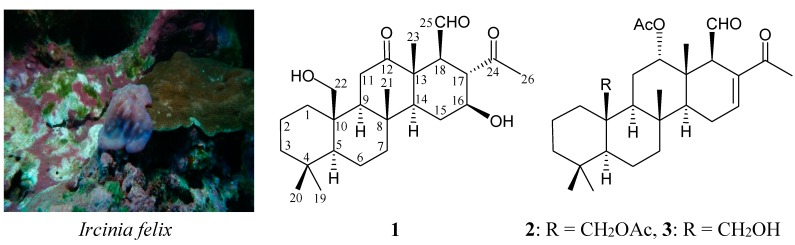
The sponge *Ircinia felix* and the structures of felixins F (**1**), G (**2**) and 12α-acetoxy-22-hydroxy-24-methyl-24-oxoscalar-16-en-25-al (**3**).

The sponge *Ircinia felix* (Duchassaing and Michelotti, 1864) (family Irciniidae, order Dictyoceratida, class Demospongiae, phylum Porifera) (Figure 1) has been proven to be an important source of interesting natural substances [1,2,3,4,5], and the extract from this organism has also played interesting roles in marine ecology [6,7,8,9,10] and medicinal use [11,12]. In our previous study, five new scalarane analogues, felixins A–E, were isolated from *I. felix* [13], and several compounds showed cytotoxicity. Scalarane-type sesterterpenoids were proven to be active in a number of bioassays, particularly in cytotoxic activity, and played important roles in chemical markers and chemical ecology [14]. In the further study of this interesting organism, two new 24-homoscalarane analogues, felixins F (**1**) and G (**2**), were isolated (Figure 1). In this paper, we deal with the isolation, structure determination, and cytotoxicity of homoscalaranes **1** and **2**.

## 2. Results and Discussion

Felixin F (**1**) was isolated as a white powder and the molecular formula for this compound was determined to be C_26_H_40_O_5_ (seven unsaturations) using high-resolution electron spray mass spectroscopy (HRESIMS) (C_26_H_40_O_5_ + Na, *m*/*z* 455.27662, calculated 455.27680). Comparison of the ^13^C NMR and distortionless enhancement by polarization transfer (DEPT) data with the molecular formula indicated that there must be two exchangeable protons, which require the presence of two hydroxy groups. The ^13^C NMR and DEPT data showed that this compound has 26 carbons (Table 1), including five methyls, eight sp^3^ methylenes (including one oxymethylene), six sp^3^ methines (including one oxymethine), four sp^3^ quaternary carbons, and three carbonyls. Thus, from the above data, three degrees of unsaturation were accounted for and **1** was identified as a tetracyclic sesterterpenoid analogue. From the ^1^H–^1^H correlation spectroscopy (COSY) of **1** (Table 1), it was possible to establish the separate system that maps out the proton sequences from H_2_-1/H_2_-2/H_2_-3, H-5/H_2_-6/H_2_-7, H-9/H_2_-11, and H-14/H_2_-15/H-16/H-17/H-18/H-25. These data, together with the key heteronuclear multiple bond connectivity (HMBC) correlations between protons and quaternary carbons (Table 1), such as H_2_-3, H_3_-19, H_3_-20/C-4; H-9, H_2_-11, H-14, H_3_-21/C-8; H-5, H-9, H_2_-22/C-10; H_2_-11, H_3_-23/C-12; H_2_-11, H-14, H_2_-15, H-18, H_3_-23/C-13; and H-17, H_3_-26/C-24, established the main carbon skeleton of **1** as a 24-homoscalarane analogue [14]. The oxymethylene unit at δ_C_ 62.7 was correlated to the methylene protons at δ_H_ 4.07 and 3.93 in the heteronuclear multiple quantum coherence (HMQC) spectrum and these methylene signals were ^2^*J*-correlated with C-10 (δ_C_ 42.7) and ^3^*J*-correlated with C-1 (δ_C_ 33.9), C-5 (δ_C_ 56.8), and C-9 (δ_C_ 61.8), proving the attachment of a hydroxymethyl group at C-10 (Table 1).

**Table 1:** ^1^H (400 MHz, CDCl_3_) and ^13^C (100 MHz, CDCl_3_) NMR data and ^1^H–^1^H COSY and HMBC correlations for homoscalarane **1**.

Position	δ_H_ (*J* in Hz)	δ_C_, Multiple	^1^H–^1^H COSY	HMBC
1	2.09 m; 0.55 ddd (12.8, 12.8, 3.2)	33.9, CH_2_	H_2_-2	n.o.
2	1.54–1.37 m	17.8, CH_2_	H_2_-1, H_2_-3	n.o.
3	1.42 m; 1,16 m	41.5, CH_2_	H_2_-2	C-4, -20
4		33.0, C		
5	0.94 dd (12.8, 2.4)	56.8, CH	H_2_-6	C-6, -10, -20, -22
6	1.54–1.37 m	18.2, CH_2_	H-5, H_2_-7	C-5
7	1.93 m; 1.15 m	30.0, CH_2_	H_2_-6	n.o.
8		38.2, C		
9	1.28 (14.4, 2.4)	61.8, CH	H_2_-11	C-8, -10, -21, -22
10		42.7, C		
11	3.24 dd (14.4, 14.4); 2.53 dd (14.4, 2.4)	38.6, CH_2_	H-9	C-8, -9, -12, -13
12		214.6, C		
13		52.4, C		
14	1.29 m	57.3, CH	H_2_-15	C-7, -8, -13, -15, -16, -23
15	1.90 m; 1.02 m	41.9, CH_2_	H-14, H-16	C-13, -14, -16, -17
16	3.57 ddd (10.8, 10.8, 4.8)	73.3, CH	H_2_-15, H-17	n.o.
17	2.91 dd (11.6, 10.8)	53.0, CH	H-16, H-18	C-16, -18, -24
18	3.18 d (11.6)	57.2, CH	H-17, H-25	C-13, -16, -23, -25
19	0.86 s	33.5, CH_3_		C-3, -4, -5, -20
20	0.75 s	21.8, CH_3_		C-3, -4, -5, -19
21	1.26 s	16.4, CH_3_		C-8, -9, -14
22	4.07 d (11.6); 3.93 d (11.6)	62.7, CH_2_		C-1, -5, -9, -10
23	1.19 s	15.6, CH_3_		C-12, -13, -14, -18
24		212.7, C		
25	9.89 s	204.4, CH	H-18	C-13, -17, -18
26	2.37 s	33.8, CH_3_		C-17, -24

n.o. = not observed.

**Figure 2:**
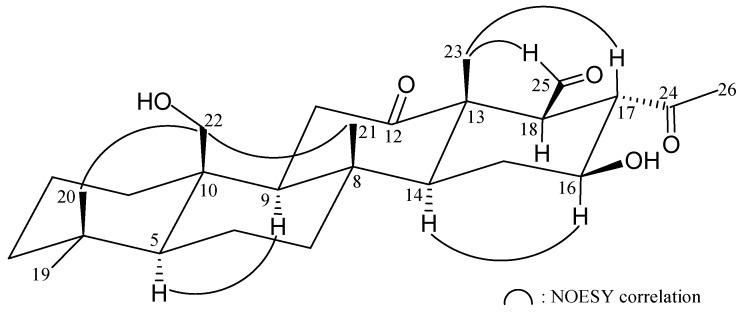
Selective NOESY correlations of **1**.

The relative stereochemistry of **1** was elucidated from the nuclear Overhauser effect (NOE) interactions observed in nuclear Overhauser effect spectroscopy (NOESY) (Figure 2). As per convention, when analyzing the stereochemistry of scalarane sesterterpenoids, H-5 and hydroxymethyl at C-10 were assigned to the α and β face, respectively, anchoring the stereochemical analysis because no correlation was found between H-5 and H_2_-22. In the NOESY experiment of **1**, H-9 showed a correlation with H-5 but not with H_3_-21 and H_2_-22. Thus, H-9 must be on the α face while Me-21 and the hydroxymethyl at C-10 must be located on the β face. Moreover, the correlations of H-14 with H-16, but not with H_3_-21 and H_3_-23, indicated the β-orientations of Me-23 and the hydroxy group attaching at C-13 and C-16, respectively. H_3_-23 showed correlations with H-17 and H-25, and large coupling constants were recorded between H-16/H-17 (*J* = 10.8 Hz) and H-17/H-18 (*J* = 11.6 Hz), indicating that the dihedral angles between H-16/H-17 and H-17/H-18 are approximately 180° and H-17 and the aldehyde group at C-18 have β-orientations. Based on the above findings, the structure of **1** was established unambiguously.

The HRESIMS of **2** (felixin G) exhibited a pseudomolecular ion peak at *m*/*z* 523.30321 [M + Na]^+^, with the molecular formula C_30_H_44_O_6_ (calcd. C_30_H_44_O_6_ + Na, 523.30301), implying nine degrees of unsaturation. The ^13^C NMR and DEPT spectra of **2** exhibited 30 carbons: one aldehyde (δ_C_ 200.8, CH-25), one ketone (δ_C_ 198.7, C-24), two ester carbonyls (δ_C_ 171.0, 169.9, 2× acetate carbonyls), one trisubstituted olefin (δ_C_ 142.6, CH-16; 137.2, C-17), one oxymethylene (δ_C_ 64.8, CH_2_-22), one oxymethine (δ_C_ 74.8, CH-12), seven methyls, seven methylenes, four methines, and four quaternary carbons. The ^1^H NMR spectrum showed seven methyls (δ_H_ 2.34, 3H, s, H_3_-26; 2.17, 2.04, 2 × 3H, each s, acetate methyls; 1.03, 3H, s, H_3_-21; 0.95, 3H, s, H_3_-23; 0.89, 3H, s, H_3_-19; 0.83, 3H, s, H_3_-20); one acetoxymethylene (δ_H_ 4.58, 1H, d, *J* = 12.0 Hz; 4.13, 1H, d, *J* = 12.0 Hz, H_2_-22); one oxymethine (δ_H_ 4.76, 1H, s, H-12); one olefinic proton (δ_H_ 7.09, 1H, dd, *J* = 2.5, 2.5 Hz, H-16); and one aldehyde proton (δ_H_ 9.41, 1H, d, *J* = 3.5 Hz, H-25). A typical 24-methylscalarane carbon system bearing acetoxymethylene and four methyl groups along rings A–D could be established by the HMBC correlations from the acetoxymethylene (CH_2_-22) and four methyl groups (Me-19, -20, -21, and 23) to the associated carbons and a 24-homoscalarane skeleton could be obtained on the basis of further HMBC and ^1^H–^1^H COSY correlations (Table 2).

**Table 2:** ^1^H (500 MHz, CDCl_3_) and ^13^C (125 MHz, CDCl_3_) NMR data and ^1^H–^1^H COSY and HMBC correlations for homoscalarane **2**.

Position	δ_H_ (*J* in Hz)	δ_C_, Multiple	^1^H–^1^H COSY	HMBC
1	1.98 m; 0.53 ddd (12.5, 12.5, 3.0)	34.7, CH_2_	H_2_-2	C-3
2	1.56 m; 1.41 m	18.1, CH_2_	H_2_-1, H_2_-3	C-1, -10
3	1.44 m; 1.18 m	41.5, CH_2_	H_2_-2	C-2, -19, -20
4		32.9, C		
5	0.99 dd (17.0, 4.0)	56.8, CH	H_2_-6	C-4, -6, -10, -20, -22
6	1.54 m; 1.44 m	17.9, CH_2_	H-5, H_2_-7	C-5, -8
7	1.88 m; 1.18 m	41.9, CH_2_	H_2_-6	C-8, -9
8		37.8, C		
9	1.39 m	51.9, CH	H_2_-11	C-1, -8, -10, -11, -12, -14, -21, -22
10		40.1, C		
11	2.15–2.05 m	24.2, CH_2_	H-9, H-12	n.o.
12	4.76 s	74.8, CH	H_2_-11	n.o.
13		40.0, C		
14	1.52 m	49.2, CH	H_2_-15	C-9, -15, -23
15	2.26–2.30 m	23.7, CH_2_	H-14, H-16	C-16, -17
16	7.09 dd (2.5, 2.5)	142.6, CH	H_2_-15	n.o.
17		137.2, C		
18	3.53 broad s	53.0, CH	H-25	n.o.
19	0.89 s	33.7, CH_3_		C-3, -4, -5, -20
20	0.83 s	21.9, CH_3_		C-3, -4, -5, -19
21	1.03 s	16.1, CH_3_		C-7, -8, -9, -14
22	4.58 d (12.0); 4.13 d (12.0)	64.8, CH_2_		C-1, -9, -10, acetate carbonyl
23	0.95 s	15.2, CH_3_		C-12, -13, -14, -18
24		198.7, C		
25	9.41 d (3.5)	200.8, CH	H-18	C-18
26	2.34 s	25.1, CH_3_		C-24
12-OAc		169.9, C		
	2.17 s	21.2, CH_3_		Acetate carbonyl
22-OAc		171.0, C		
	2.04 s	21.5, CH_3_		Acetate carbonyl

n.o. = not observed.

**Figure 3:**
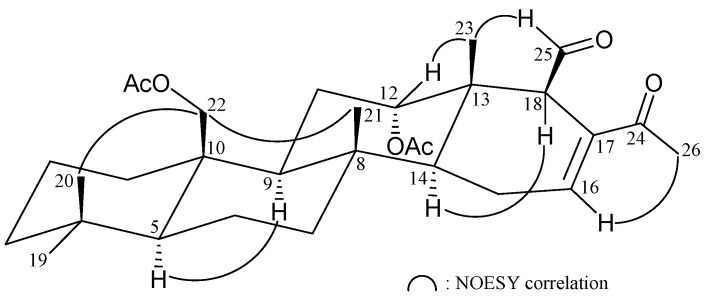
Selective NOESY correlations of **2**.

The relative stereochemistry of **2** was elucidated from the interactions observed in a NOESY experiment (Figure 3). In the NOESY experiment of **2**, H-9 showed a correlation with H-5, but not with H_3_-21 and H_2_-22. Thus, H-5 and H-9 must be on α face while Me-21 and the acetoxymethylene at C-10 must be located on the β face. H-14 correlated with H-18, but not with H_3_-21 and H_3_-23, assuming that H-14 and H-18 were α-oriented. The correlation of H_3_-23 with H-12, but not with H-14, indicated the β-orientations of Me-23 and H-12. H-16 showed a correlation with H_3_-26, revealing the *E* geometry of the C-16/17 double bond. It was found that the structure of **2** was similar with that of a known 24-homoscalarane analogue, 12α-acetoxy-22-hydroxy-24-methyl-24-oxoscalar-16-en-25-al (**3**) [15], except that the 22-hydroxy group in **3** was replaced by an acetoxy group in **2**.

The cytotoxicity of homoscalaranes **1** and **2** against CCRF-CEM (human acute lymphoblastic leukemia), HL-60 (human acute promyelocytic leukemia), K-562 (human chronic myelogenous leukemia), MOLT-4 (human acute lymphoblastic leukemia), SUP-T1 (human T-cell lymphoblastic lymphoma), U-937 (human histiocytic lymphoma), DLD-1 (human colorectal adenocarcinoma), LNCaP (human prostatic carcinoma), and MCF7 (human breast adenocarcinoma) tumor cells is shown in Table 3. Compound **1** was found to show cytotoxicity toward the leukemia K562, MOLT-4, and SUP-T1 cells (IC_50_ ≤ 5.0 μM). By comparing the cytotoxic data of **1** with those of **2** and the relative scalarane derivatives, flexins A−E, that we isolated previously [13], we find that **1** is more cytotoxic toward most tumor cells.

**Table 3:** Cytotoxic data of homoscalaranes **1** and **2***.*

Compounds	Cell Lines IC_50_ (μM)								
	CCRF-CEM	HL-60	K-562	MOLT-4	SUP-T1	U-937	DLD-1	LNCaP	MCF7
**1**	NT ^a^	NT	1.27	2.59	3.56	10.65	19.26	7.22	NT
**2**	7.90	6.50	19.9	NT	NT	13.08	27.08	17.14	NA ^b^
Doxorubicin ^c^	0.02	0.02	0.70	0.02	0.09	0.33	0.90	3.16	0.29

^a^ NT = not test; ^b^ NA = not active at 20 μg/mL for 72 h; ^c^ Doxorubicin was used as a positive control.

## 3. Experimental Section

### 3.1. General Experimental Procedures

Optical rotation values were measured with a Jasco P-1010 digital polarimeter (Japan Spectroscopic Corporation: Tokyo, Japan). IR spectra were obtained on a Jasco FT-IR 4100 spectrophotometer (Japan Spectroscopic Corporation); absorptions are reported in cm^−1^. NMR spectra were recorded on a Varian Mercury Plus 400 NMR spectrometer (Varian Inc.: Palo Alto, CA, USA) or a Varian Inova 500 spectrometer (Varian Inc.) using the residual CHCl_3_ signal (δ_H_ 7.26 ppm) as the internal standard for ^1^H NMR and CDCl_3_ (δ_C_ 77.1 ppm) for ^13^C NMR. Coupling constants (*J*) are given in Hz. ESIMS and HRESIMS were recorded using a Bruker 7 Tesla solariX FTMS system (Bruker: Bremen, Germany). Column chromatography was performed on silica gel (230–400 mesh; Merck: Darmstadt, Germany). TLC was carried out on precoated Kieselgel 60 F_254_ (0.25 mm; Merck: Darmstadt, Germany); spots were visualized by spraying with 10% H_2_SO_4_ solution followed by heating. Normal phase HPLC (NP-HPLC) was performed using a system comprised of a Hitachi L-7110 pump (Hitachi Ltd.: Tokyo, Japan) and a Rheodyne 7725 injection port (Rheodyne LLC: Rohnert Park, CA, USA). A normal phase column (Supelco Ascentis^®^ Si Cat #: 581515-U, 25 cm × 21.2 mm, 5 μm; Sigma-Aldrich: St. Louis, MO, USA) was used for HPLC.

### 3.2. Animal Material

Specimens of the sponge *Ircinia felix* (Duchassaing and Michelotti, 1864) [16] were collected by hand using self-containing underwater breathing apparatus (SCUBA) equipment off the coast of the Southern Taiwan (Johnson Outdoors Inc.: Racine, WI, USA), on 5 September 2012, and stored in a freezer until extraction. A voucher specimen (NMMBA-TWSP-12005) was deposited in the National Museum of Marine Biology & Aquarium, Pingtung, Taiwan.

### 3.3. Extraction and Isolation

Sliced bodies of *Ircinia felix* (wet weight 1210 g) were extracted with ethyl acetate (EtOAc). The EtOAc layer (5.09 g) was separated on silica gel and eluted using a mixture of *n*-hexane and EtOAc (stepwise, 100:1–pure EtOAc) to yield 11 fractions A–K. Fraction H was chromatographed on silica gel and eluted using *n*-hexane/acetone (6:1–2:1) to afford 14 fractions H1–H14. Fraction H2 was separated by NP-HPLC using a mixture of dichloromethane (DCM) and EtOAc (5:1, flow rate: 2.0 mL/min) to afford **2** (1.4 mg, *t*_R_ = 121 min). Fraction I was separated by NP-HPLC using a mixture of dichloromethane (DCM) and acetone (4:1, flow rate: 2.0 mL/min) as the mobile phase to yield **1** (1.8 mg, *t*_R_ = 81 min).

Felixin F (**1**): white solid; mp 117−120 °C; ${\text{[α]}}_{\text{D}}^{25}$ +54 (*c* 0.4, CHCl_3_); IR (neat) ν_max_ 3462, 1704 cm^−1^; ^1^H (400 MHz, CDCl_3_) and ^13^C (100 MHz, CDCl_3_) NMR data, see Table 1; ESIMS: *m*/*z* 455 [M + Na]^+^; HRESIMS: *m*/*z* 455.27662 (calcd. for C_26_H_40_O_5_ + Na, 455.27680).

Felixin G (**2**): white solid; mp 121−124 °C; ${\text{[α]}}_{\text{D}}^{25}$ +43 (*c* 0.3, CHCl_3_); IR (neat) ν_max_ 1738 cm^−1^; ^1^H (500 MHz, CDCl_3_) and ^13^C (125 MHz, CDCl_3_) NMR data, see Table 2; ESIMS: *m*/*z* 523 [M + Na]^+^; HRESIMS: *m*/*z* 523.30321 (calcd. for C_30_H_44_O_6_ + Na, 523.30301).

### 3.4. MTT Antiproliferative Assay

CCRF-CEM, HL-60, K-562, MOLT-4, SUP-T1, U-937, DLD-1, LNCaP, and MCF7 cells were obtained from the American Type Culture Collection (ATCC; Manassas, VA, USA). Cells were maintained in RPMI 1640 medium supplemented with 10% fetal calf serum, 2 mM glutamine, and antibiotics (100 units/mL penicillin and 100 μg/mL streptomycin) at 37 °C in a humidified atmosphere of 5% CO_2_. Cells were seeded at 4 × 10^4^ per well in 96-well culture plates before treatment with different concentrations of the tested compounds. The compounds were dissolved in dimethyl sulfoxide (less than 0.02%) and made concentrations of 1.25, 2.5, 5, 10, and 20 μg/μL prior to the experiments. After treatment for 72 h, the cytotoxicity of the tested compounds was determined using a MTT cell proliferation assay (thiazolyl blue tetrazolium bromide, Sigma-M2128, St. Louis, MO, USA). The MTT is reduced by the mitochondrial dehydrogenases of viable cells to a purple formazan product. The MTT-formazan product was dissolved in DMSO. Light absorbance values (OD = OD_570_ − OD_620_) were recorded at wavelengths of 570 and 620 nm using an ELISA reader (Anthos labtec Instrument, Salzburg, Austria) to calculate the concentration that caused 50% inhibition (IC_50_), *i.e.*, the cell concentration at which the light absorbance value of the experiment group was half that of the control group. These results were expressed as a percentage of the control ± SD established from *n* = 4 wells per one experiment from three separate experiments [17,18,19].

## 4. Conclusions

Our further studies on *Ircinia felix* for the extraction of natural substances have led to the isolation of five new 20-homoscalaranes, felixins F (**1**) and G (**2**) and compound **1** are potentially cytotoxic toward the leukemia K562, MOLT-4, and SUP-T1 cells. These results suggest that continuing investigation of novel secondary metabolites together with the potentially useful bioactivities from *I. felix* are worthwhile for future drug development.
